# Inhibition of Pancreatic Lipase by Flavonoid Derivatives: *In Vitro* and *In Silico* Investigations

**DOI:** 10.1155/2024/6655996

**Published:** 2024-01-24

**Authors:** The-Huan Tran, Thanh-Tan Mai, Thi-Thu-Trang Ho, Thi-Ngoc-Dung Le, Thi-Cam-Nhung Cao, Khac-Minh Thai, Thai-Son Tran

**Affiliations:** ^1^Faculty of Pharmacy, Hue University of Medicine and Pharmacy, Hue University, Hue 530000, Vietnam; ^2^Faculty of Pharmacy, University of Medicine and Pharmacy at Ho Chi Minh City, Ho Chi Minh 700000, Vietnam; ^3^Faculty of Pharmacy, Hue Medical College, Hue 530000, Vietnam

## Abstract

Obesity, characterized by excessive adipose tissue accumulation, has emerged as a crucial determinant for a wide range of chronic medical conditions. The identification of effective interventions for obesity is of utmost importance. Widely researched antiobesity agents focus on pancreatic lipase, a significant therapeutic target. This study presented the evaluation of ten flavonoid compounds in terms of their inhibitory activities against pancreatic lipase, utilizing both *in vitro* and *in silico* approaches. The results indicated that all tested compounds demonstrated modest and weaker inhibitory activities compared to the reference compound, orlistat. Among the compounds investigated, F01 exhibited the highest potency, with an IC_50_ value of 17.68 ± 1.43 *µ*M. The enzymatic inhibition kinetic analysis revealed that F01 operated through a competitive inhibition mechanism with a determined *K*_*i*_ of 7.16 *μ*M. This value suggested a moderate binding affinity for the pancreatic lipase enzyme. Furthermore, the associated *V*_max_ value was quantified at 0.03272 ΔA·min^−1^. *In silico* studies revealed that F01 displayed a binding mode similar to that of orlistat, despite lacking an active functional group capable of forming a covalent bond with Ser152 of the catalytic triad. However, F01 formed a hydrogen bond with this crucial amino acid. Furthermore, F01 interacted with other significant residues at the enzyme's active site, particularly those within the lid domain. Based on these findings, F01 demonstrates substantial potential as a candidate for further investigations.

## 1. Introduction

Over the past four decades, there has been a significant and concerning escalation in the global prevalence of obesity, posing a substantial challenge to public health. Extensive investigations conducted by the World Health Organization (WHO) have revealed a remarkable upward trajectory, with the proportion of individuals classified as obese nearly tripling since 1975. Obesity, characterized by the excessive accumulation of adipose tissue, has emerged as a pivotal determinant for a diverse range of chronic medical conditions, thereby amplifying the burden on both individuals and healthcare systems [1]. Notably, a pronounced association exists between obesity and type 2 diabetes, necessitating focused attention [2]. However, the implications of obesity extend beyond diabetes, as compelling evidence consistently demonstrates a significant correlation between obesity and numerous cardiovascular diseases [3]. Furthermore, the incidence of obesity exhibits a close relationship with various neoplastic pathologies [4], including breast [5], colorectal [6], kidney [7], and pancreatic [8] cancers, thus emphasizing the urgent need to address this global health crisis. In addition to its ramifications on physical health, obesity profoundly affects mental well-being [9]. Scientific investigations consistently elucidate a robust association between obesity and mental health issues, such as depression [10], anxiety [11], and low self-esteem [12]. Recent times have witnessed the emergence of interactions between obesity and infectious diseases, particularly in the context of the ongoing COVID-19 pandemic [13]. Obesity adversely impacts respiratory function, compromising lung capacity and rendering individuals more susceptible to severe respiratory infections [14]. Considering the exacerbating influence of obesity on the potential consequences of severe illness and mortality, there is an urgent imperative to identify efficacious interventions for obesity.

In addition to other approaches, a key objective in obesity treatment is to identify inhibitors of pancreatic lipase that can effectively diminish the breakdown and absorption of nutrients [15]. Inhibiting the activity of pancreatic lipase and subsequently decreasing fat absorption presents a promising therapeutic strategy for addressing obesity [16]. Orlistat currently represents the sole pancreatic lipase inhibitor approved for clinical use, demonstrating the capacity to reduce fat absorption by approximately 30% in dietary contexts [17]. However, despite its efficacy, the clinical application of orlistat is linked to mild to moderate gastrointestinal side effects. Consequently, ongoing research efforts are dedicated to the exploration and development of novel pancreatic lipase inhibitors that possess improved safety profiles and diminished adverse effects for individuals with obesity.

Flavonoids, a class of natural compounds widely distributed in plants, exhibit a remarkable range of biological activities, making them of great interest in scientific research. Their potential benefits in promoting human health have garnered significant attention, particularly in the context of various disease conditions [18]. Numerous studies have shed light on the potential therapeutic effects of flavonoids in diverse areas, including cardiovascular disorders [19], neurodegenerative diseases [20], certain types of cancer [21], osteoporosis [22], obesity [23], and diabetes [24]. The multifaceted nature of flavonoids allows them to interact with various molecular targets and cellular processes, thereby exerting beneficial effects on human physiology. In the context of obesity, flavonoids have shown promise in modulating metabolic pathways and contributing to weight management [23]. They may exert antiobesity effects by influencing adipogenesis, lipid metabolism, and energy expenditure [25]. Given the wide array of potential effects associated with flavonoids, this particular study aimed to evaluate the inhibitory activity of specific flavonoid derivatives against pancreatic lipase. By identifying and evaluating specific flavonoid derivatives that exhibit potent inhibitory activity against pancreatic lipase, this study sought to identify promising candidates for further development as antiobesity agents.

## 2. Experimental Setup

### 2.1. Materials and Equipment

#### 2.1.1. Materials

**Figure 1:**
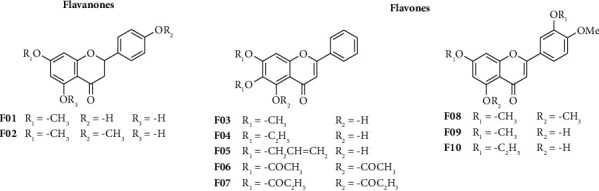
Structure of flavonoid derivatives used in this study.

Ten semisynthetic flavonoid derivatives, 2 flavanones and 8 flavones (Figure 1), reported in the previous works were used in this study [26, 27]. Porcine pancreatic lipase, type II (L-3126); substrate of p-nitrophenyl palmitate (p-NPP); orlistat; and other chemicals were purchased from Merck Millipore (Burlington, Massachusetts, United States) and Sigma-Aldrich (St. Louis, Missouri, United States).

#### 2.1.2. Equipment

Clinical Microplate Reader Touch Screen EMR-500 (Labomed Inc., Los Angeles, United States) and refrigerated Centrifuge Z206A (Hermle Labortechnik, Wehingen, Germany) were used. Data are processed using Microsoft Excel 2021 software (Microsoft, Washington, United States) and GraphPad Prism 8.4.3 software (GraphPad Software, Massachusetts, United States). All computation processes were performed on a computer system with the processor of Intel® CoreTM i7-7700 CPU @ 3.60 Hz, 16.0 GB of RAM, the Visual Graphic Card of NVIDIA GeForce GT 1030 2 GB, and the operating system of 64 bit Windows 10 (Microsoft, Washington, United States).

### 2.2. Investigation of Enzyme Inhibitory Activity

The pancreatic lipase inhibition assays were conducted using the spectrophotometric method with p-nitrophenyl palmitate (p-NPP) as the substrate and orlistat as a reference compound. Following the experimental conditions described in the previous study [28], the assay was performed as follows: each sample solution, composed of 50 mM Tris-HCl buffer at pH 8.0, tested compound, and 10 mg/ml pancreatic lipase enzyme, was sequentially added. The resulting mixture was thoroughly mixed and incubated for 10 minutes at 37°C. Subsequently, the p-NPP substrate solution was added and mixed well. The mixture was then further incubated for 7 minutes at 37°C. The absorbance of the solution was measured at 405 nm. For the control solution, the same procedure was followed, replacing the tested compound with 10% DMSO. The blank solution was prepared with no enzyme added.

The percentage of pancreatic lipase inhibition (*I*%) was calculated using the following equation:

(1) $$\begin{array}{c} I\%=\left[\frac{\left(∆A_{0}-∆A\right)}{A_{0}}\right]\times 100. \end{array}$$

Here, ∆*A*_0_ represents the absorbance difference between the control and the blank, ∆*A* represents the absorbance difference between the sample and the blank, and *A*_0_ represents the absorbance of the control.

A linear regression equation was constructed to establish the correlation between the logarithm of the concentration (*µ*M) of the tested substances and the percentage of pancreatic lipase inhibition. From this equation, the IC_50_ value, which indicates the concentration of the compound required for 50% inhibition, was determined.

### 2.3. Enzymatic Inhibition Kinetic Analysis

The kinetic evaluation of pancreatic lipase enzyme activity involved the selection of the most potent pancreatic lipase inhibitor, along with the inclusion of the positive control agent, orlistat. The substrate p-NPP was examined at five distinct concentrations ranging from 12.5 *μ*M to 200 *μ*M. The flavonoid derivative was assessed for its lipase inhibition capacity over time at concentrations of 15 *μ*M and 30 *μ*M. Simultaneously, a negative control sample without the inhibitor was utilized for comparison. Similarly, the control substance orlistat underwent testing at concentrations of 0.1 *μ*M and 0.2 *μ*M. Inhibition constants (*K*_*i*_) and maximum velocity (*V*_max_) were derived from the Michealis–Menten plots, and the mode of enzyme inhibition was elucidated through the Lineweaver–Burk plots. All parameters and graphical representations were calculated and generated using GraphPad Prism 8.4.3 software.

### 2.4. Molecular Docking

The molecular docking investigation employed methodologies and software outlined in a previous publication [27]. Ligand preparation, docking procedures, and interaction analysis were carried out using Sybyl X 2.0 (Certara, New Jersey, United States), FlexX (BioSolveIT GmbH, North Rhine-Westphalia, Germany), and MOE 2008.10 (Chemical Computing Group, Montreal, Canada) computational programs, respectively. Default settings were utilized for these processes. In this study, the 1LPB protein complex, derived from the Protein Data Bank [29], was selected. This complex comprises the pancreatic lipase-colipase inhibited by a C11 alkyl phosphonate, and its structure was obtained at a resolution of 2.46 angstroms. To validate the docking protocols, a pose selection approach was employed [30]. This involved comparing the root-mean-square deviation (RMSD) value between the redocked conformations and the original ligand bound in the cocrystallized complex. A value of ≤1.5 Å served as a criterion for confirming the reliability of the docking models.

### 2.5. Molecular Dynamics Simulations

Molecular dynamics (MDs) simulations were conducted using GROMACS 2021 [31] to investigate the binding between an inhibitor and the enzyme. The protein's topology was generated within GROMACS 2021 using the CHARMM27 force field [32]. The most favorable conformation of the inhibitor was saved as a *∗*.mol2 file, which served as the foundation for generating the ligand's topology. This process was accomplished using the SwissParam server (https://www.swissparam.ch) [33] along with the same force field. To create a suitable environment, the protein-ligand complex was placed in a dodecahedral box with a minimum distance of 1 nm maintained between the complex and the box edges. Subsequently, a water solvent (TIP3P model) was added, and Na^+^ or Cl^−^ ions were introduced to achieve neutralization (NaCl concentration of 0.15 M). Energy minimization of the system was carried out for 100 ps to alleviate any clashes or unfavorable conformations. This was followed by NVT and NPT equilibration processes lasting 100 ps each, during which the system was adjusted to a temperature of 300 K and a pressure of 1 bar. The MDs process was then executed for 100 ns, saving each frame at 10 ps intervals for subsequent analysis. To evaluate the MDs results, parameters, such as root-mean-square deviation (RMSD) and root-mean-square fluctuation (RMSF), were employed to assess the stability of the protein and ligand. Additionally, solvent-accessible surface area (SASA) and radius of gyration (Rg) values were calculated for the protein-ligand complex. The binding of the ligand to the protein was assessed by analyzing the frequency of hydrogen bond formation and water-mediated interactions. Hydrogen bonds were identified using a cutoff distance of ≤3.5 Å and an angle of ≤120°, utilizing the VMD 1.9.3 software [34]. The MOE 2008.10 software, along with the Protein Ligand Interaction Fingerprints (PLIFs) tool, was utilized to quantify the occurrence of water-mediated interactions between the protein and ligand.

## 3. Results and Discussion

### 3.1. Pancreatic Lipase Inhibition Assay

Table 1 presents the results of evaluating the inhibitory activity of ten flavonoid derivatives and orlistat against pancreatic lipase. The results indicate that the biological activities of the investigated compounds were rather modest. All compounds exhibited weaker pancreatic lipase inhibition compared to orlistat, with most of them having IC_50_ values exceeding 100 *µ*M (except for F01 and F02). Notably, among the derivatives, F01 (5-hydroxy-2-(4-hydroxyphenyl)-7-methoxychroman-4-one) displayed the highest potency with an IC_50_ value of 17.68 ± 1.43 *µ*M. Orlistat, a well-known reference compound, was employed as a control in this activity assessment, as it is frequently utilized in studies investigating pancreatic lipase inhibition. Analysis of the IC_50_ values in conjunction with the structural characteristics of the derivatives suggests that flavonoids bearing a flavanone structure (F01 and F02) seem to exhibit superior inhibitory activities against pancreatic lipase compared to flavones (F03–F10).

### 3.2. Enzymatic Inhibition Kinetic Analysis

**Figure 2:**
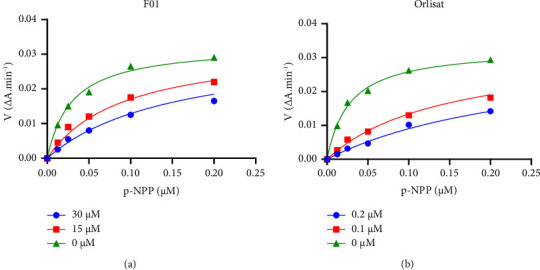
Michealis–Menten plot for F01 (a) and orlistat (b).

Figure 2 illustrates the correlation between substrate concentration and the hydrolysis rate of the lipase enzyme in the presence of either inhibitor F01 (Figure 2(a)) or orlistat (Figure 2(b)) at various concentrations. The plot reveals that, as substrate concentration increases, competition arises, diminishing the inhibitor's capacity to bind to the enzyme. At sufficiently high substrate concentrations, the enzyme's hydrolysis rate reaches *V*_max_, akin to the scenario without an inhibitor. Moreover, *V*_max_ values in the presence of F01 and orlistat are comparable, measuring 0.03272 and 0.03295 ΔA·min^−1^, respectively. This underscores that both compounds exert their inhibitory effects on pancreatic lipase enzyme through a competitive inhibition mechanism. The inhibition constant (*K*_i_) of F01 was determined to be 7.16 *μ*M, indicative of a moderate binding affinity for the pancreatic lipase enzyme. Conversely, orlistat demonstrated a robust binding affinity to pancreatic lipase, with a value of *K*_i_ of 0.02 *μ*M. These findings substantiate that both F01 and orlistat employ competitive inhibition as their mode of action, elucidating the varying degrees of binding affinities exhibited by the two compounds toward the pancreatic lipase enzyme.

**Figure 3:**
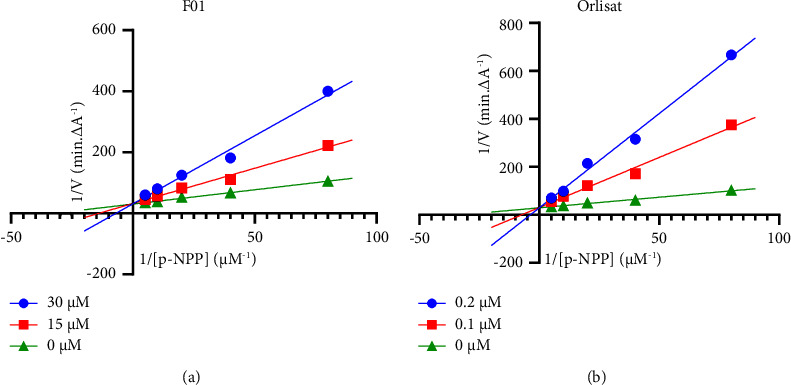
Lineweaver–Burk plot for F01 (a) and orlistat (b).

The Lineweaver–Burk plot for compound F01 (Figure 3(a)) demonstrates an intersection on the vertical axis, signifying competitive inhibition of the pancreatic lipase enzyme. Similar competitive inhibitory characteristics against pancreatic lipase are observed in certain other flavonoid compounds, consistent with the behavior exhibited by F01 [35–37]. Notably, orlistat, as depicted in Figure 3(b), also manifested competitive inhibition against the pancreatic lipase enzyme, aligning with previously reported findings on the inhibitory properties of this compound [38].

### 3.3. Molecular Docking

**Figure 4:**
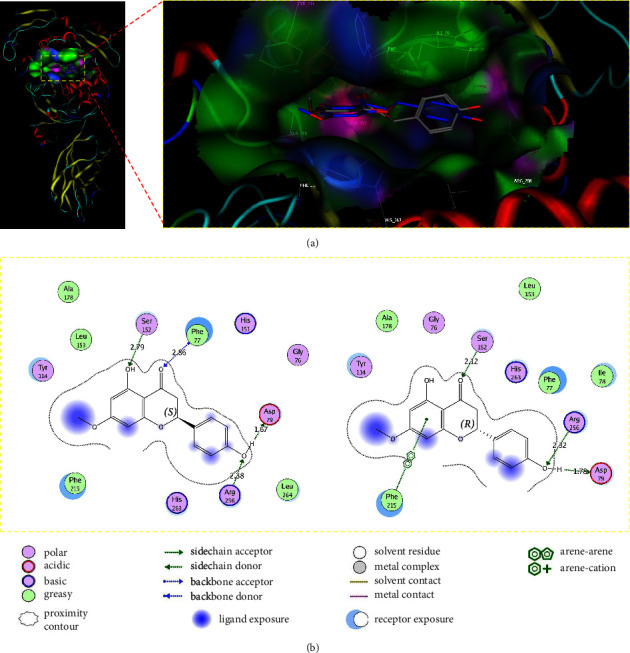
The 3D representation (panel (a)) and 2D depiction (panel (b)) reveal the ligand interaction of F01 within the binding site. In panel (a), (*S*)-F01 is presented with gray carbon atoms, while (*R*)-F01 is depicted with blue carbon atoms. In panel (b), the lengths of hydrogen bonds are expressed in angstroms.

**Figure 5:**
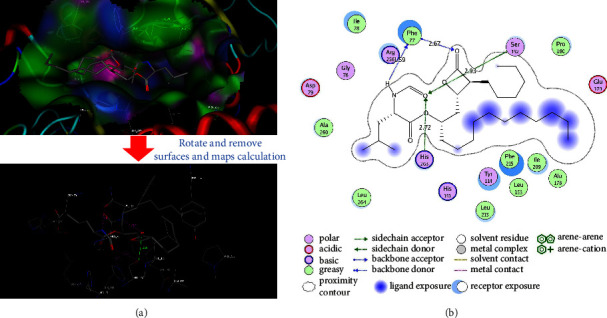
Ligand interaction of orlistat within the binding site obtained from molecular docking study presented in 3D (panel (a)) and 2D (panel (b)) depictions. The lengths of hydrogen bonds are expressed in angstroms.

The findings from the molecular docking study are presented in Figures 4 and 5 and supplementary material (Table S1). The results indicate that both the (*S*) and (*R*) enantiomers of F01 exhibited a similar binding mode to the target. In particular, (*S*)–F01 interacted with specific residues at the binding site through *van der Waals* forces, including Gly76, Ile78, Tyr114, Leu153, Ala178, Phe215, His263, and Leu264. Furthermore, (*S*)–F01 formed four hydrogen bonds with critical residues: Ser152 (length: 2.31 Å), Phe77 (length: 2.80 Å), Asp79 (length: 1.48 Å), and Arg256 (length: 2.27 Å). Similarly, (*R*)-F01 interacted with Gly76, Phe77, Ile78, Tyr114, Ala178, Leu153, and His263 through *van der Waals* forces at the binding site. It also formed three hydrogen bonds with Ser152 (length: 2.12 Å), Asp79 (length: 1.78 Å), and Arg256 (length: 2.32 Å). Moreover, (*R*)-F01 engaged in an arene-arene interaction with Phe215 at the binding site. Notably, Gly76, Phe77, Ile78, Asp79, and Phe215 are residues located in the lid domain, whereas Ser152 is an essential amino acid within the catalytic triad (Ser152-Asp176-His263) on the enzyme [39]. Consequently, the binding characteristics of F01 to the binding site closely resemble those of orlistat. For orlistat, its *β*-lactone warhead has been found to exhibit reversible covalent inhibition on the active site Ser152, while its long hydrophobic chains interact with residues within the lid domain, specifically Gly76 to Phe80 and Leu213 to Met217 of pancreatic lipase [40]. Computational findings derived from a molecular docking investigation for orlistat (depicted in Figure 5) revealed a pronounced resemblance in the mode of interaction between orlistat and the enzyme when compared to experimental observations [40]. Specifically, the active *β*-lactone ring of orlistat demonstrated close proximity to the OH group of Ser152 within the enzyme, maintaining a distance of approximately 2.55 Å (Figure 5(a)). This spatial arrangement fostered favorable conditions for the establishment of a covalent bond. Furthermore, the hydrophobic chains of orlistat exhibited significant interactions with the residues residing in the lid domain (Figure 5(b)).

In this study, the F01 derivative displayed an IC_50_ value of 17.68 ± 1.43 *µ*M, which was lower than that of orlistat (IC_50_ = 0.14 ± 0.00 *µ*M). This discrepancy could be attributed to the F01 derivative's rigid structure and the absence of an active functional group capable of forming a covalent bond with Ser152. Among all the substances examined in this study, F01 stood out as the sole compound capable of interacting with Arg256. The significance of this interaction with Arg256 for potential pancreatic lipase inhibitory activity was highlighted by Sridhar et al. in their previous studies [41, 42]. Consequently, the observed ability of F01 to engage with Arg256 might explain its superior potency in inhibiting pancreatic lipase compared to the other substances evaluated in this study.

### 3.4. Molecular Dynamics Simulation

#### 3.4.1. Enzyme Stability

**Figure 6:**
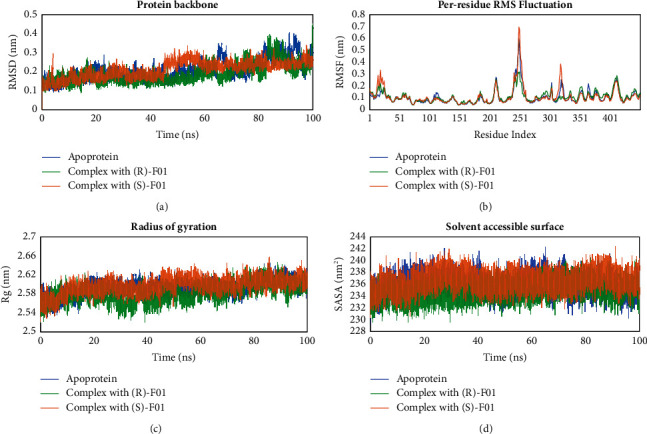
Stability of pancreatic lipase in apoprotein form and in complex with F01 during 100 ns of molecular dynamics simulations. (a) RMSD values of protein carbon backbone, (b) RMSF value of each residue of the enzyme, (c) Radius of protein gyration, and (d) Solvent accessible surface area of the enzyme.

The stability of the pancreatic lipase in its apoprotein and complexed form with F01 could be observed in Figure 6(a). The RMSD values of the protein carbon backbone indicate that the complex was more stable than the apoprotein. During the 0–45 ns period, the motion of the apoprotein and the complex was quite similar to each other. However, from 45 to 100 ns, the complex with (*S*) and (*R*)-enantiomers achieved higher stability than the apoprotein (with average RMSD values of 0.244 ± 0.044 nm, 0.214 ± 0.051 and, 0.236 ± 0.056 nm, respectively). The RMSD fluctuation of the complex at the end of the simulation period was also only about 0.1 nm. For the residues in the binding site, the average fluctuation between the complex and the apoprotein forms was not significant (Figure 6(b)). Similarly, Figures 6(c) and 6(d) show that other characteristics of the pancreatic lipase such as the radius of protein gyration and solvent accessible surface area also did not change significantly between the complex and apoprotein form (the average Rg for complex form with (*S*)-F01 = 2.597 ± 0.017 nm, Rg for complex form with (*R*)-F01 = 2.582 ± 0.019 nm, Rg for apoprotein = 2.593 ± 0.015 nm; SASA for complex form with (*S*)-F01 = 236.5 ± 1.5 nm^2^, SASA for complex form with (*R*)-F01 = 234.9 ± 1.5 nm^2^, SASA for apoprotein = 236.0 ± 1.6 nm^2^).

#### 3.4.2. Stability of Ligand and Its Interaction with the Enzyme

**Figure 7:**
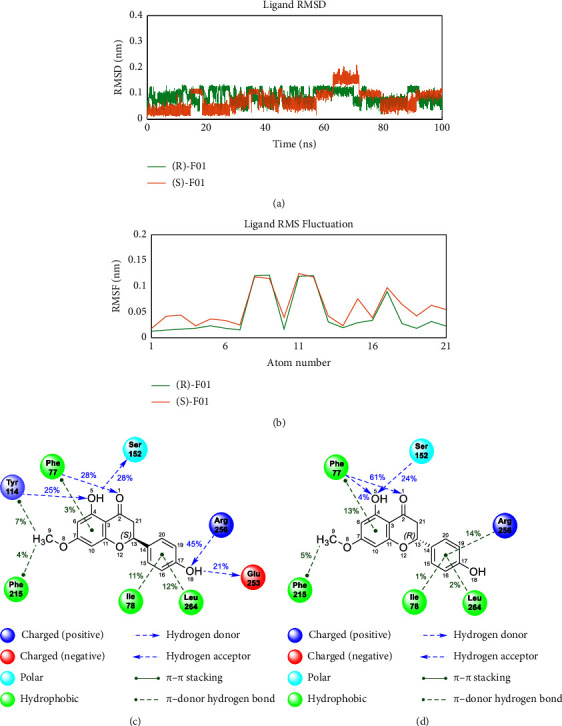
Ligand stability and its interactions with pancreatic lipase. (a) RMSD values of ligand's heavy atoms during 100 ns of molecular dynamics simulations. (b) RMSF value of each heavy atoms of the ligand. (c, d) Schematic of protein-ligand interactions analyzed using data from 100 ns MDs trajectory.

During the MDs process, the ligand showed a definite degree of flexibility as presented in Figure 7(a), but the RMSD magnitude did not exceed 0.2 nm. Initially, the (*S*)-enantiomer had an average RMSD value of approximately 0.058 nm in the first 60 ns, followed by a conformational change leading to an average RMSD value of 0.121 nm from 60 to 80 ns, which then quickly settled back to an average RMSD value of 0.073 nm in the remaining time. Concurrently, the (*R*)-enantiomer manifested heightened stability, as indicated by an average ligand RMSD value of 0.089 ± 0.023 nm throughout the entire duration of the simulation, spanning 100 nanoseconds.

The average displacement of each of the F01's heavy atoms was calculated and presented in Figure 7(b). In general, all of the ligand's heavy atoms had RMSF values of less than 0.2 nm. However, some atoms with higher-than-average displacement could be observed such as O8, C9, C11, C12, C15, and C17. The important protein-ligand interactions were analyzed, including hydrogen bonds and hydrophobic interactions. The hydrogen bonds between (*S*)-F01 and the Phe77, Ser152, and Arg256 amino acids in the docking structure were found to persist during the MDs process with frequencies of 28, 28, and 45%, respectively. Specifically, Phe77 formed a hydrogen bond with the O1 atom, Ser152 accepted a hydrogen bond from the -O5H group, and Arg256 formed a hydrogen bond (*S*) with the O18 atom. Additionally, (S)-F01 also formed hydrogen bonds with other residues in the binding sites such as Tyr114 and Glu253 with frequencies of 25 and 21%, respectively. Ring A of (*S*)-F01 formed a *π*-*π* stacking interaction with Phe77, while Ring B formed a similar interaction with Ile78 and Leu264. The methoxy group on ring A also played its role by forming a *π*-donor hydrogen bond with Tyr114 and Phe215 (Figure 7(c)). Figure 7(d) illustrates that (*R*)-F01 exhibited enzyme interactions highly comparable to those of (*S*)-F01. Crucial interactions with amino acid residues Ser152, Phe215, and Arg256 were largely preserved throughout the molecular dynamics simulations. Furthermore, (*R*)-F01 demonstrated interactions with Phe77, Ile78, and Leu264 akin to those observed with (*S*)-F01.

## 4. Conclusions

This study aimed to assess the inhibitory activity of ten flavonoid compounds against pancreatic lipase through *in vitro* experimentation. The results demonstrated that all the compounds under investigation displayed relatively moderate and weaker inhibitory activity when compared to the reference compound, orlistat. Notably, among the compounds examined, F01 exhibited the most potent inhibition of pancreatic lipase, as evidenced by its IC_50_ value of 17.68 ± 1.43 *µ*M. The enzymatic inhibition kinetics analysis revealed that F01 was acting as a competitive inhibitor with a *K*_i_ of 7.16 *μ*M. This value suggested a moderate binding affinity for the pancreatic lipase enzyme. Furthermore, the associated *V*_max_ value was quantified at 0.03272 ΔA·min^−1^. *In silico* studies further elucidated that F01 adopted a binding mode similar to that of orlistat, despite lacking a functional group capable of forming a covalent bond with Ser152 of the catalytic triad. Nonetheless, F01 established a hydrogen bond with this critical residue, indicating its capacity to interact with the enzyme. Moreover, F01 exhibited interactions with other significant amino acids within the binding site of the enzyme, particularly those associated with the lid domain. While this study did not identify any substance exhibiting superior biological activity compared to orlistat, the absence of such superiority does not rule out the potential of a substance to function as an effective drug. It is crucial to recognize that various factors, such as the specific binding site and mode of interaction, significantly contribute to the pharmacological effects of a compound. Consequently, F01 exhibits considerable promise as a potential candidate for subsequent investigations.

## Figures and Tables

**Table 1:** IC_50_ values of flavonoid derivatives for pancreatic lipase.

Compounds^*∗*^	IC_50_ (*µ*M)
**F01**	**17.68** **±** **1.43**
**F02**	**76.22** **±** **3.22**
F03	>>500
F04	>>500
F05	340.51 ± 24.10
F06	>>500
F07	>>500
F08	269.89 ± 14.55
F09	464.63 ± 11.76
F10	>>500
Orlistat	0.14 ± 0.00

^*∗*^The structures of flavonoid derivatives are presented in Figure 1. The studied compounds with the highest biological activity are highlighted in bold.

## Data Availability

The data used to support the findings of this study are included within the article.

## Supplementary Materials

Supplementary Materials Table S1: results of molecular docking studies of flavonoid derivatives on pancreatic lipase (protein complex: 1LPB).
