# Supplementary material for: Inhibition of Pancreatic Lipase by Flavonoid Derivatives: In Vitro and In Silico Investigations
Source: Adv Pharmacol Pharm Sci. 2024 Jan 24;2024:6655996. doi: 10.1155/2024/6655996 (PMC10830309; doi:10.1155/2024/6655996)
Supplement: Supplementary Materials — Table S1: results of molecular docking studies of flavonoid derivatives on pancreatic lipase (protein complex: 1LPB). [file 6655996.f1.docx]

**Inhibition of pancreatic lipase by flavonoid derivatives: *in vitro* and *in silico*** **investigations**

The-Huan Tran^1^, Thanh-Tan Mai^2^, Thi-Thu-Trang Ho^1^, Thi-Ngoc-Dung Le^3^,
Thi-Cam-Nhung Cao^1^, Khac-Minh Thai^2^, Thai-Son Tran^1^*

*^1^Faculty of Pharmacy, Hue University of Medicine and Pharmacy, Hue University, Hue City 530000, Vietnam*

*^2^Faculty of Pharmacy, University of Medicine and Pharmacy at Ho Chi Minh City, Ho Chi Minh City 700000, Vietnam*

*^3^Faculty of Pharmacy, Hue Medical College, Hue City 530000, Vietnam*

* To whom correspondence should be addressed:

Thai-Son Tran

Email: [tthaison@hueuni.edu.vn](mailto:tthaison@hueuni.edu.vn)

Orcid:

The-Huan Tran: 0000-0003-2949-3665

Thanh-Tan Mai: 0000-0001-7313-9853

Thi-Cam-Nhung Cao: 0000-0002-1701-8149

Khac-Minh Thai: 0000-0002-5279-9614

Thai-Son Tran: 0000-0002-3023-7792

**Supplementary material**

**Table S1. Results of molecular docking studies of flavonoid derivatives on pancreatic lipase (protein complex: 1LPB)**

| No. | Compound | Ligand interaction |
| --- | --- | --- |
| 1 | *(S)-*F02 | 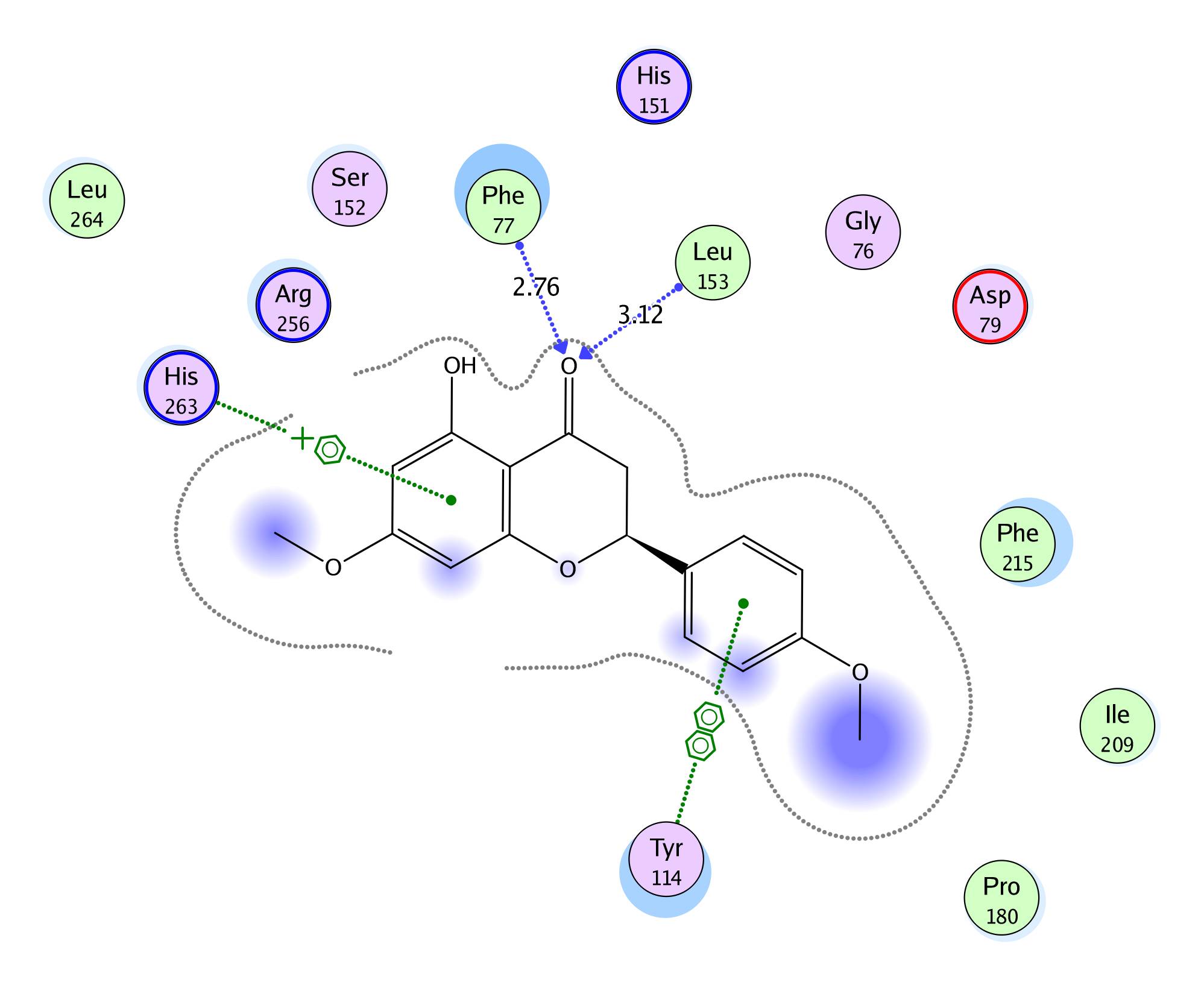 |
| 2 | *(R)-*F02 | 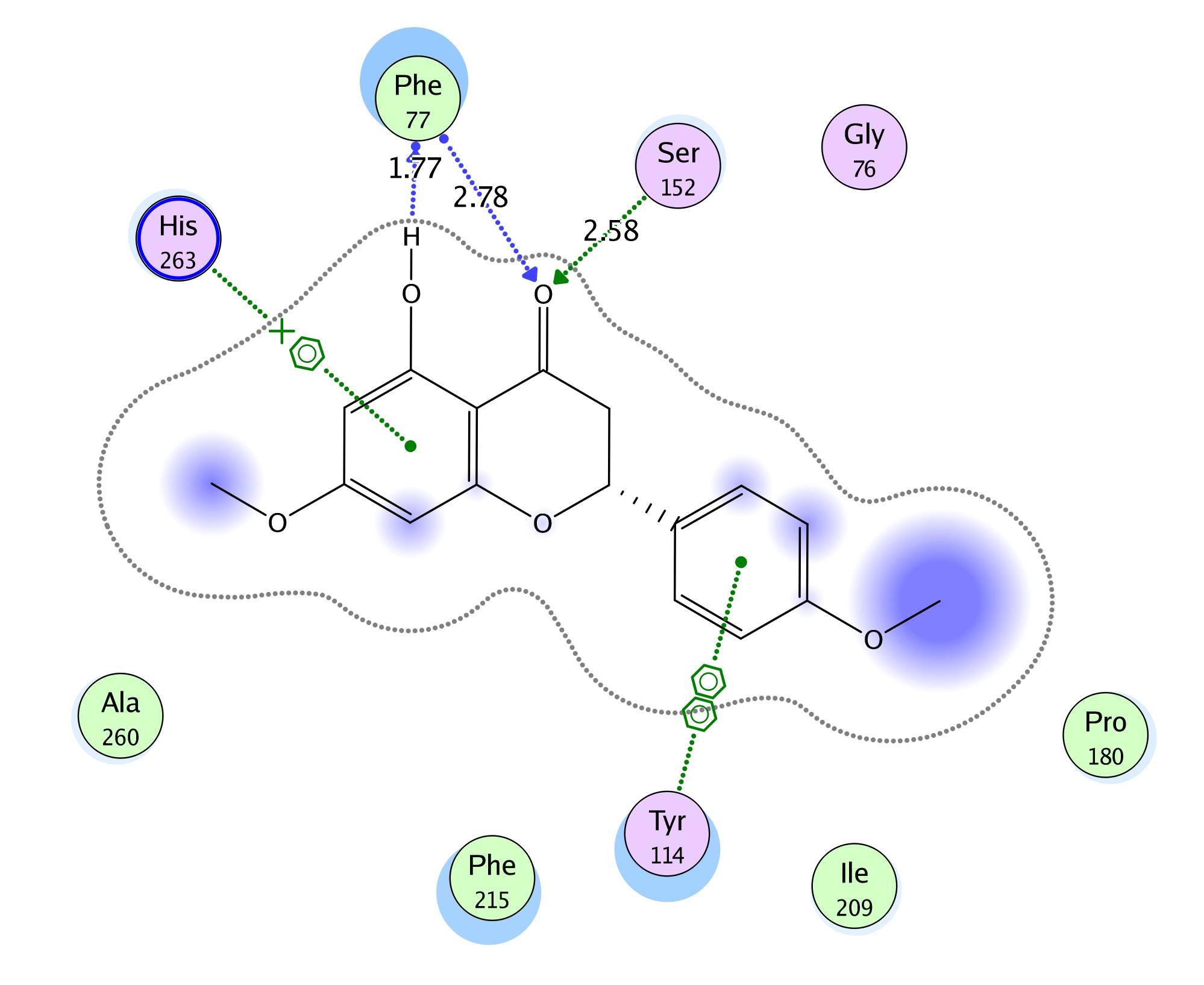 |
| 3 | F03 | 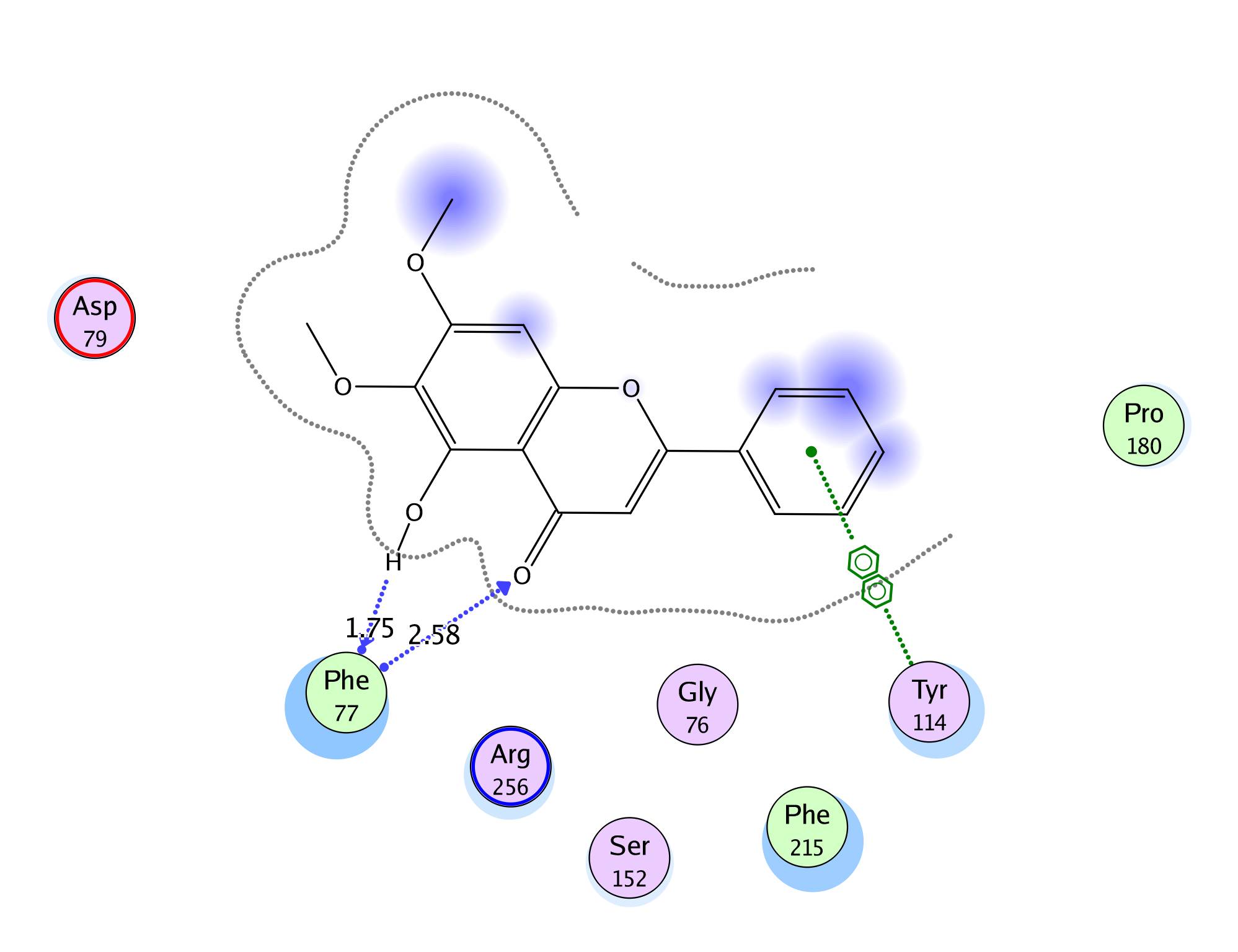 |
| 4 | F04 | 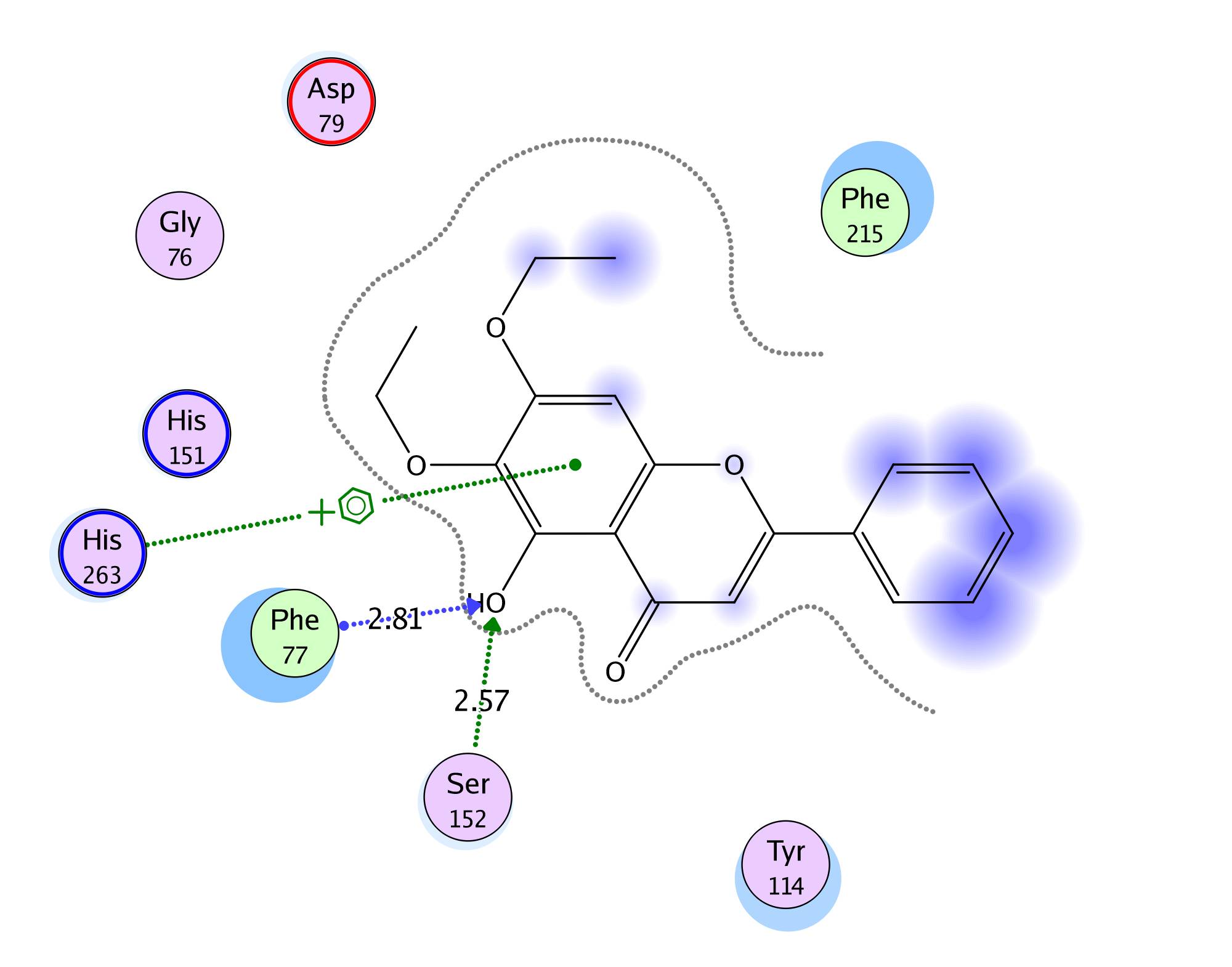 |
| 5 | F05 | 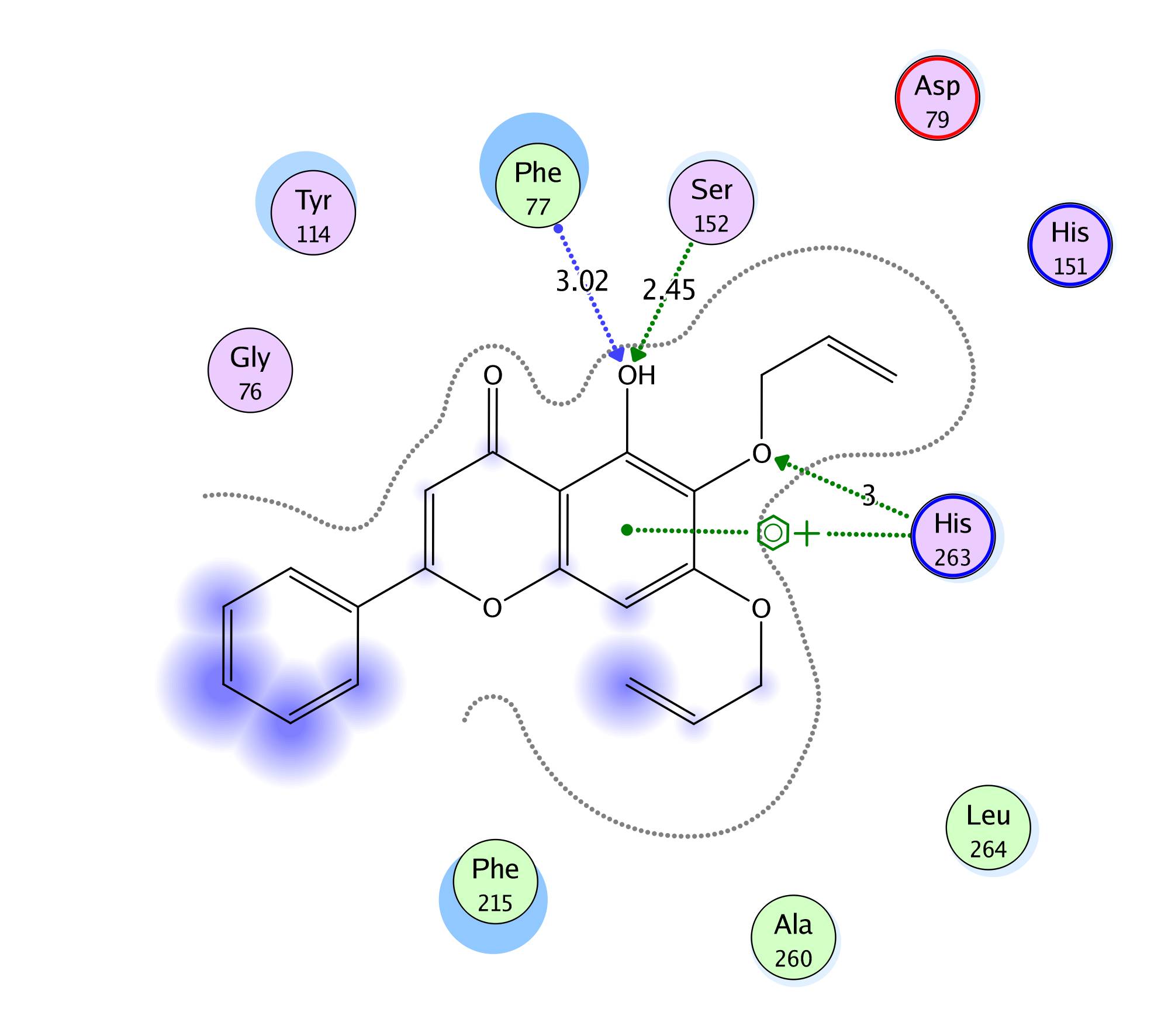 |
| 6 | F06 | 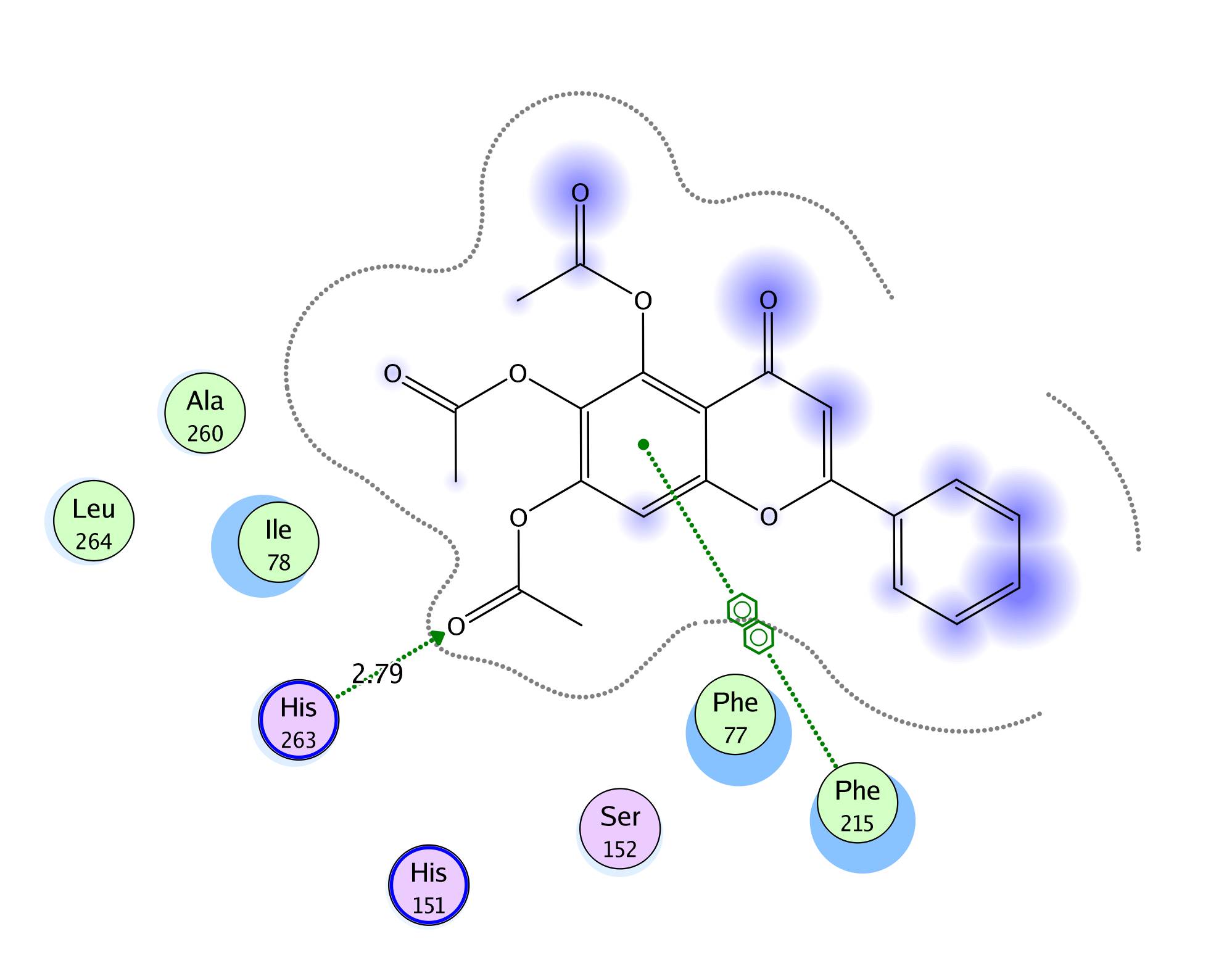 |
| 7 | F07 | 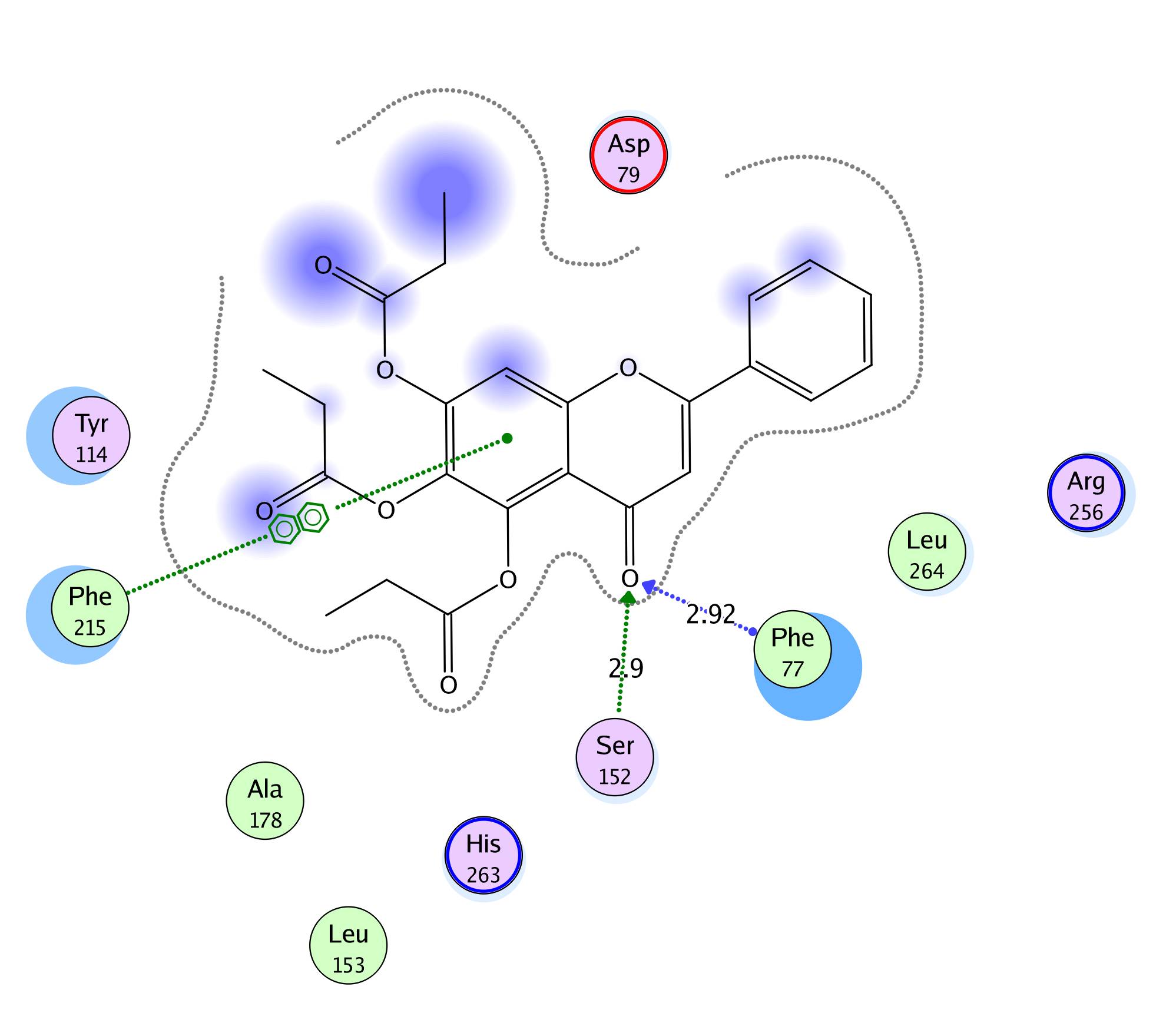 |
| 8 | F08 | 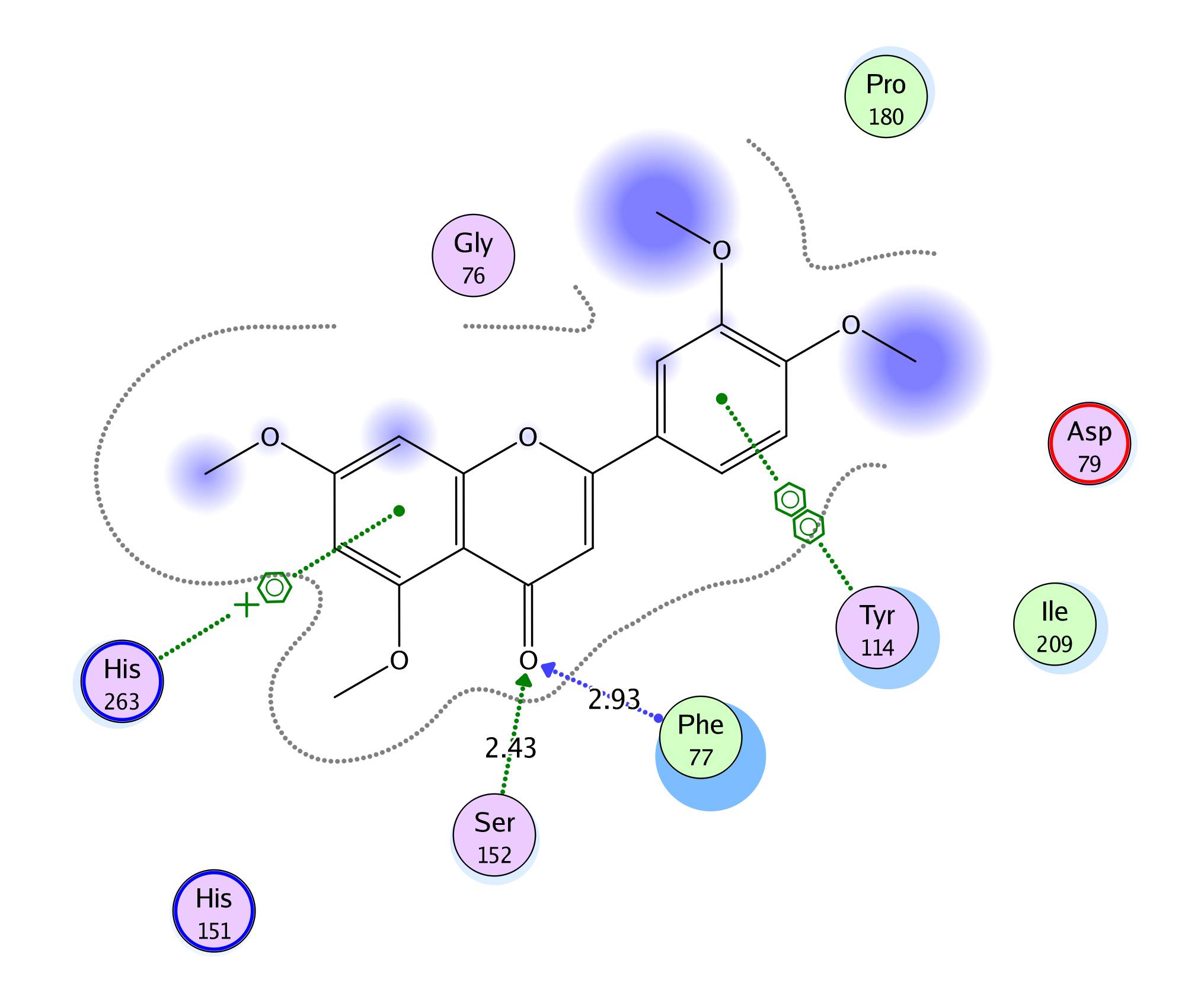 |
| 9 | F09 | 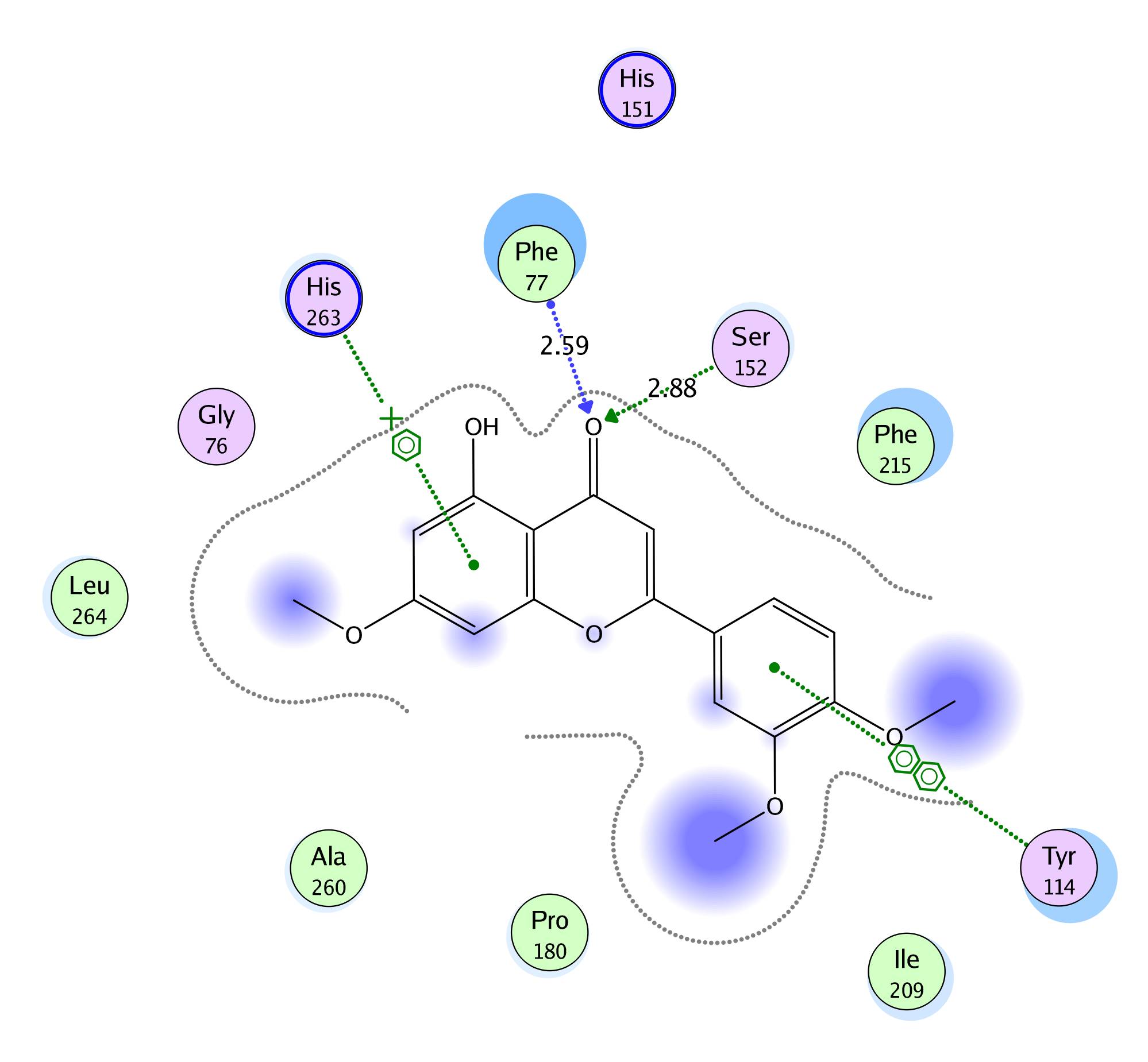 |
| 10 | F10 | 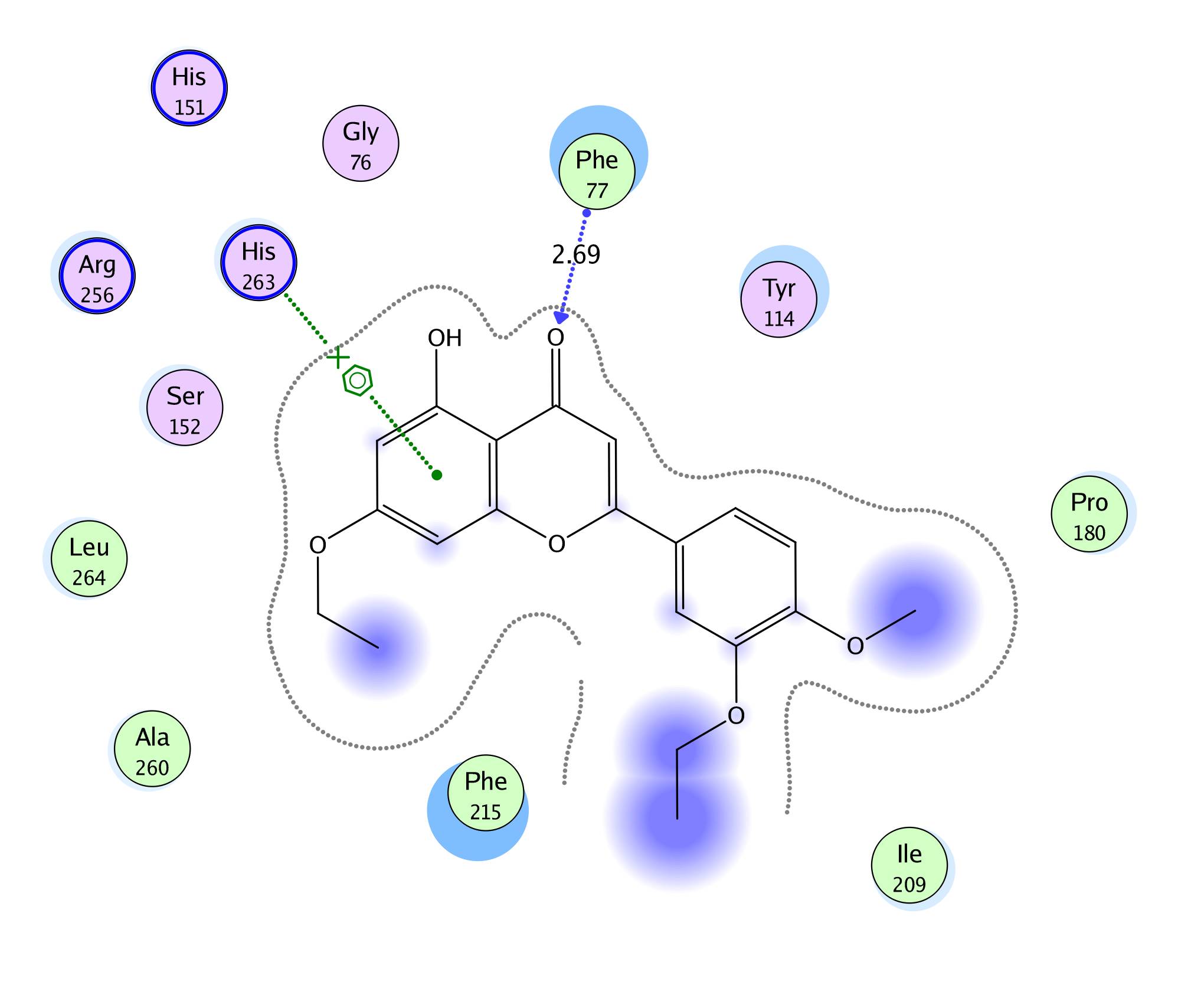 |
| 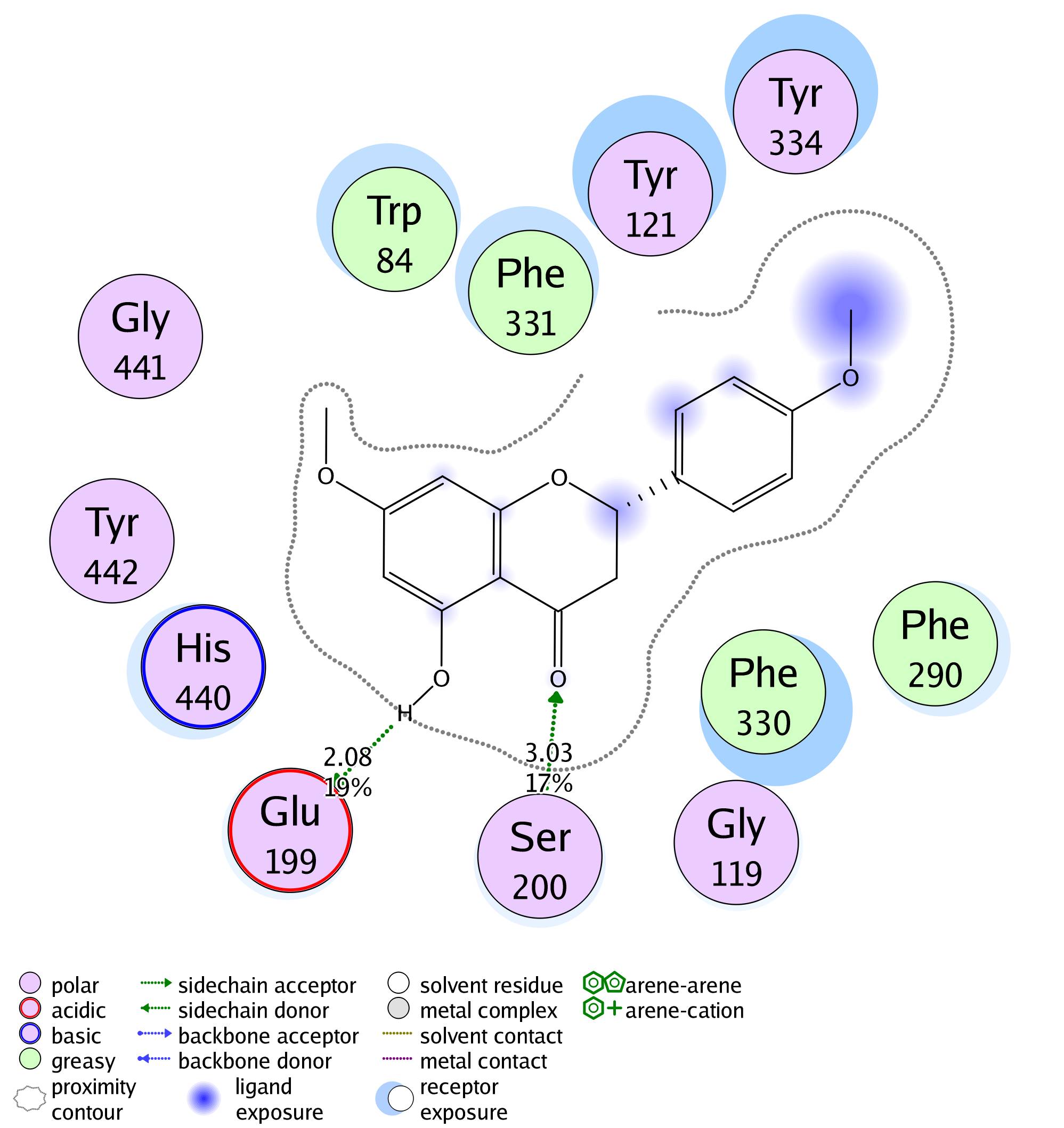 | | |

** The lengths of hydrogen bonds are expressed in angstroms*
